# DNA aptamer-based rolling circle amplification product as a novel immunological adjuvant

**DOI:** 10.1038/s41598-020-79420-w

**Published:** 2020-12-17

**Authors:** Adil S. Al-Ogaili, Rohana Liyanage, Jack O. Lay, Tieshan Jiang, Christine N. Vuong, Shilpi Agrawal, Thallapuranam Krishnaswamy Suresh Kumar, Luc R. Berghman, Billy M. Hargis, Young Min Kwon

**Affiliations:** 1 Department of Cell and Molecular Biology, University of Arkansas, Fayetteville, AR 72701 USA; 2 Department of Medical Laboratory Techniques, Kut Tech Institute, Middle Technical University, Baghdad, Iraq; 3 Chemistry and Biochemistry, University of Arkansas, Fayetteville, AR 72701 USA; 4 Statewide Mass Spectrometry Facility, Fayetteville, AR 72701 USA; 5 Department of Poultry Science, University of Arkansas, Fayetteville, AR 72701 USA; 6 Department of Poultry Science and Pathobiology, Texas A&M University, College Station, TX 77843 USA

**Keywords:** Vaccines, Biotechnology, Immunology, Molecular medicine

## Abstract

Several agonists to CD40 have shown to induce acquired immune responses. Here, we developed and evaluated the rolling circle amplification (RCA) products that are based on anti-CD40 DNA aptamers as a novel vaccine adjuvant. First, we developed DNA aptamers with specific binding affinity to chicken CD40 extra domain (chCD40ED). Next, we prepared the RCA products that consist of these aptamers to increase the spanning space and overall binding affinity to chCD40ED. Using 8 DNA aptamer candidates, 4 aptamer-based RCA products (aptamer RCAs) were generated, each consisting of two distinct aptamers. We demonstrated that all 4 aptamer RCAs significantly induced the signal transduction in chicken HD11 macrophage cell line (p < 0.05). Finally, we conjugated one of the aptamer RCAs (Aptamer RCA II) to M2e epitope peptide of influenza virus as a model hapten, and the immune complex was injected to chickens. Aptamer RCA II stimulated anti-M2e IgG antibody production to the level significantly higher as compared to the control (M2e epitope alone; p < 0.05). The results of our work suggest that aptamer RCA is a novel platform to boost the efficacy of vaccines, which might find broad applications to other antigens beyond M2e epitope evaluated in this study using chicken infection model.

## Introduction

Cluster of differentiation 40 (CD40) is a costimulatory receptor that is expressed on antigen presenting cells (APCs) and regulates acquired immune responses. The ligand for the CD40 receptor (CD40L/CD154) is transiently expressed by activated CD4^+^ T helper (T_H_) lymphocytes^1–5^. In a classical adaptive immunity, four signals are required for optimal response. Signal 0 comes from the innate immune response events when pattern recognition receptors (PRRs) attract cognate highly conserved pathogen-associated molecular patterns (PAMPs) with subsequent events such as chemokine signaling and engulfment of foreign peptide by immature dendritic cell (DC) at the periphery^6,7^. Accordingly, the DC starts the process of maturation, with processing and presenting the foreign peptide on major histocompatibility complex class II (MHC class II) molecule. In addition, maturing DC exhibits changes in cytokine production profile with concurrence homing to T zone at the draining lymph nodes. At this point, the DC is with less phagocytic capacity and more presenting activity. Signal 1 is represented by the engagement of the naïve T cell receptor and processed peptide on cognate MHC class II molecule on the DC. This engagement activates T cell and renders it express CD40L transiently with other costimulatory molecules and cytokines. The crosslinking of these costimulatory molecules represents signal 2. This will lead to downstream cell signaling in both DC and T cell. Cytokines from the DC such interleukin (IL)-1 family and IL-2 serve as the signal 3 for T cell differentiation. Similarly, activated T cell produces cytokines that affect the same cell or neighboring cells such as IL-2 and IL-4. The type of the T cell cytokines depends on the type of the foreign peptide that was initially presented by DC. The antigen-specific signal 1 is inefficient to stimulate full differentiation of naïve T cell into effector T_H_ cells (subsets), regulatory or memory T cells. T cell needs all the signals to complete proliferation and subsets differentiation such as T_H_1 or T_H_2^8–11^. When B cells are involved in antigen presentation, CD40-CD40L pathway is essential in T-cell-dependent B-cell activation. Moreover, CD40 participates in T-lymphocyte-dependent antibody class switching, antibody affinity maturation, development of memory B cells and formation of the germinal centers^12–16^.

Recent studies showed the use of different agonists to CD40 leads to induction of enhanced immune responses. The agonists, such as anti-mouse CD40 monoclonal antibodies (mAbs) or soluble CD40L, have shown to mimic the outcome of the naturally occurring CD40-CD40L interaction. Recently, our group has demonstrated that the use of CD40 mAb conjugated with hapten was significantly efficient in inducing robust immune responses in chickens^17–19^. However, the use mAb as immunological adjuvants is hindered due to high immunogenicity of mAbs themselves that might interfere with the vaccine action, and difficult and costly production. Hence, the development of alternative biomolecules that are biologically functional yet with low-immunogenicity would be more desirable for practical in vivo applications.

Aptamers, artificially prepared oligonucleotide molecules that can selectively bind to small molecular ligands, have drawn the attention as an alternative to mAbs in many biological applications with multiple advantages. Each RNA or single-strand DNA (ssDNA) molecule can have its own three-dimensional structure according to its sequence. This folding pattern creates a molecular structure to which the ligand can bind with high affinity and specificity. This property enables aptamers to have high affinities and specificities to target molecules that may exceed those of antibodies to the same target molecules. In addition, aptamers are identified via in vitro selection and characterization without requiring animals for production. The other characteristics of aptamers include thermos-stability, chemical resistance, reproducibility and low immunogenicity. Typically, aptamers can target a variety of biological structures, including proteins, viruses, and bacteria, with high affinity and specificity. Aptamers are now widely utilized for therapeutic applications, studying biological pathways or developing biomarkers^20–26^.

In this study, for the first part of the study, we developed DNA aptamers by systemic evolution of ligands by exponential enrichment (SELEX) procedure^20,21^. Then, pairs of the aptamer candidates were used produce rolling circle amplification (RCA) products^27–29^. These RCA products, termed aptamer-based RCA (aptamer RCA), consist of thousands of tandemly repeated sequences of the selected aptamers to mimic the naturally occurring receptor-ligand binding more closely. We tested these aptamer RCAs on chicken macrophage HD11 cells to monitor the stimulation via nitric oxide liberation using Griess assay^30^. With significant activation of the cell line by all 4 aptamer RCAs tested, we sought to test if these aptamer RCAs can serve as vaccine adjuvants in chickens. Aptamer RCA II, which showed strongest stimulation, was conjugated with a hapten to form an adjuvant-antigen immunizing complex. We chose M2e peptide of influenza virus as the model antigen for the immunization, since we have used this same antigen for evaluation of previous mAb-adjuvant immunizing complex and this small peptide has low immunogenicity performance. Our in vivo experimental design included subcutaneous injection of broiler chickens with two doses (25 μg/bird and 50 μg/bird) of Aptamer RCA II, which was given two times for boost immunization. The results showed that our novel immunizing complex induced robust immune response as early as 7 days post injection with the higher dose and with both doses after 21 days. The novel adjuvant we developed in the form of aptamer RCA may find broad applications in conjunction with other antigens targeted by APCs.

## Results and discussion

### Activation of chicken HD11 macrophage cell line via CD40 receptor by DNA aptamer-based rolling circle amplification products

The naturally occurring CD40/CD40L interaction is an essential process in both branches of any given immune response. In recent studies, our group demonstrated a novel anti-chCD40 mAb (designated mAb 2C5) has the ability to induce significant stimulation in chicken HD11 macrophage cell line. When this CD40 agonist was administered using different routes in combination with a desired antigenic peptide, the CD40-targeted immunogen induced robust immune response and several desired effects such as immunoglobulin class switching occured^17–19^. However, the high cost and difficulty for mAb production would make it impractical to commercialize this as a vaccine adjuvant. Therefore, as an effort to develop an alternative solution, we sought to develop and evaluate DNA aptamers in place of mAb as an agonist to the CD40 receptor to enhance immune response.

### In vitro selection of DNA aptamers

The SELEX procedure for selection of aptamers against chicken CD40 receptor extra domain started with random ssDNA library (Supplementary Table S1) and continued for 10 enrichment cycles as described elsewhere^20,21^. Following each enrichment cycle, PCR was carried out using the resulted aptamers as the template to amplify the enriched aptamers for the next round. To resume the SELEX process, the amplified dsDNA was then digested with λ exonuclease to produce ssDNA aptamers. To enrich aptamers with increasingly higher affinity to the target protein, the molar ratio of the protein to ssDNA library was shifted gradually over the cycles by using 25% less amount of target protein in every next round^31^.

### Characterization of selected aptamers by cloning and sequencing

To evaluate the selection process, DNA products from the enrichment cycle 6 was cloned and sequenced with Sanger sequencing method^32^. One aptamer sequence (SEQ1) was repeated two times among the 15 clones sequenced and another one (SEQ2) showed 60% homology with SEQ1 (Supplementary Table S2). These aptamer sequences were analyzed by UNAFold software (Integrated DNA Technologies) to predict the secondary structure and the free energy change (ΔG) as shown in Supplementary Figure S1.

### Characterization of enriched aptamers by Illumina sequencing

**Figure 1:**
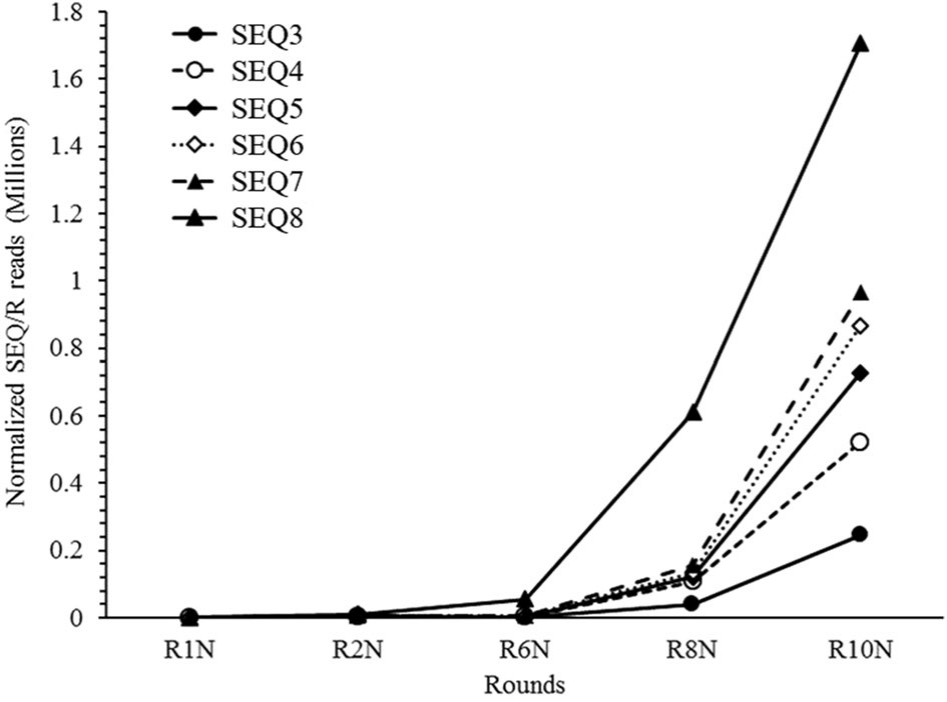
Illumina sequencing results. Normalized exponential increase of six highly enriched aptamer sequences among DNA pool over 10 rounds of the enrichment cycles of SELEX. Normalized reads were calculated using the formula: (no. of aptamer sequence reads /total no. of reads) × 100/(% the aptamer sequence in R1).

For more comprehensive characterization of the enriched aptamers, the aptamers from the cycles 1, 2, 6, 8 and 10 were sequenced using Illumina sequencing. There were around 4 × 10^6^ reads from each cycle. After quality filtering, about 95% of the row reads were retained as high-quality sequence reads. The analyzed data showed that there were six sequences (SEQ3-SEQ8; Supplementary Table S2) that were highly enriched over the SELEX enrichment cycles in the aptamer pool (Supplementary Table S3). When these six sequences were plotted against each selection rounds, SEQ8 was represented as the most abundant sequence reads, followed by SEQ7 (Fig. 1). Additionally, the predicted secondary structures of these 6 aptamers are shown in Figure S1. We performed more detailed characterization for the structure, stability and binding affinity of the two aptamers SEQ3 and SEQ4, and the results are shown in Supplementary Materials.

### Dot-blot hybridization assay

To assess the ability of the candidate aptamers to bind to the target protein, dot-blot assay was carried out with these 8 aptamer candidates (SEQ1-8). According to the visible results of the assay, all aptamer candidates demonstrated significant binding affinity to the target protein at concentration of 25 μM or higher (Supplementary Figure S2). It is important to note that the candidate aptamers evaluated here contained the primer binding sequences, due to the possibility that these sequences might be important in aptamer structure and interaction with the target protein.

### Rolling circle amplification

We speculated that the smaller size of DNA aptamers in general in comparison to the target protein might not allow adequate interactions between aptamers and CD40. To enhance the ability of the aptamers to have proper interaction with CD40, we reasoned that the RCA products that consist of these aptamer sequences might facilitate the desired interaction with the target protein. To achieve this, the reverse complimentary sequences of the eight candidate aptamer sequences were used to design 4 templates for RCA procedure with each RCA template consisting of reverse complementary sequences of two distinct aptamers (Supplementary Table S1). RCA consists of long tandemly repeated (several hundreds) single-stranded DNA sequence, which is reverse complementary of the original circular single-stranded DNA template^28,29^. Over the run of the RCA procedure, the reaction produced the RCA products of high molecular weight (Supplementary Figure S3). To confirm the molecular configuration of the aptamer RCAs, we digested the aptamer RCA II products with the restriction enzyme AgeI in the presence of the oligonucleotide with DNA sequence reverse complementary to the spacer region in the template, which contains the *Age* I recognition sequence (Spacer-complement; Supplementary Table S1). The digestion of the aptamer RCA II products with *Age* I restriction enzyme converted the RCA products of high molecular weight to a dominantly single band of the length of 195nt (Supplementary Figure S4). This result supports that the aptamer RCA products were indeed RCA products that consist of the tandemly repeated two aptamer sequences separated by spacer regions. Of note, the spacer that contains *Age* I recognition site was included in the design of the aptamer RCA for dual purposes: First, to allow efficient characterization of aptamer RCA products through digestion with *Age* I as described above. Secondly, to facilitate folding and stability of aptamer structure in the resulting aptamer RCA products by formation of the partially double stranded DNA in the spacer region with the addition of the Spacer-complement oligonucleotide. In fact, we have noticed that aptamer RCA products precipitated as aggregates in solution, which disappeared when Spacer-complement was added to the aptamer RCA products.

### Measuring the activation of macrophage cells

**Figure 2:**
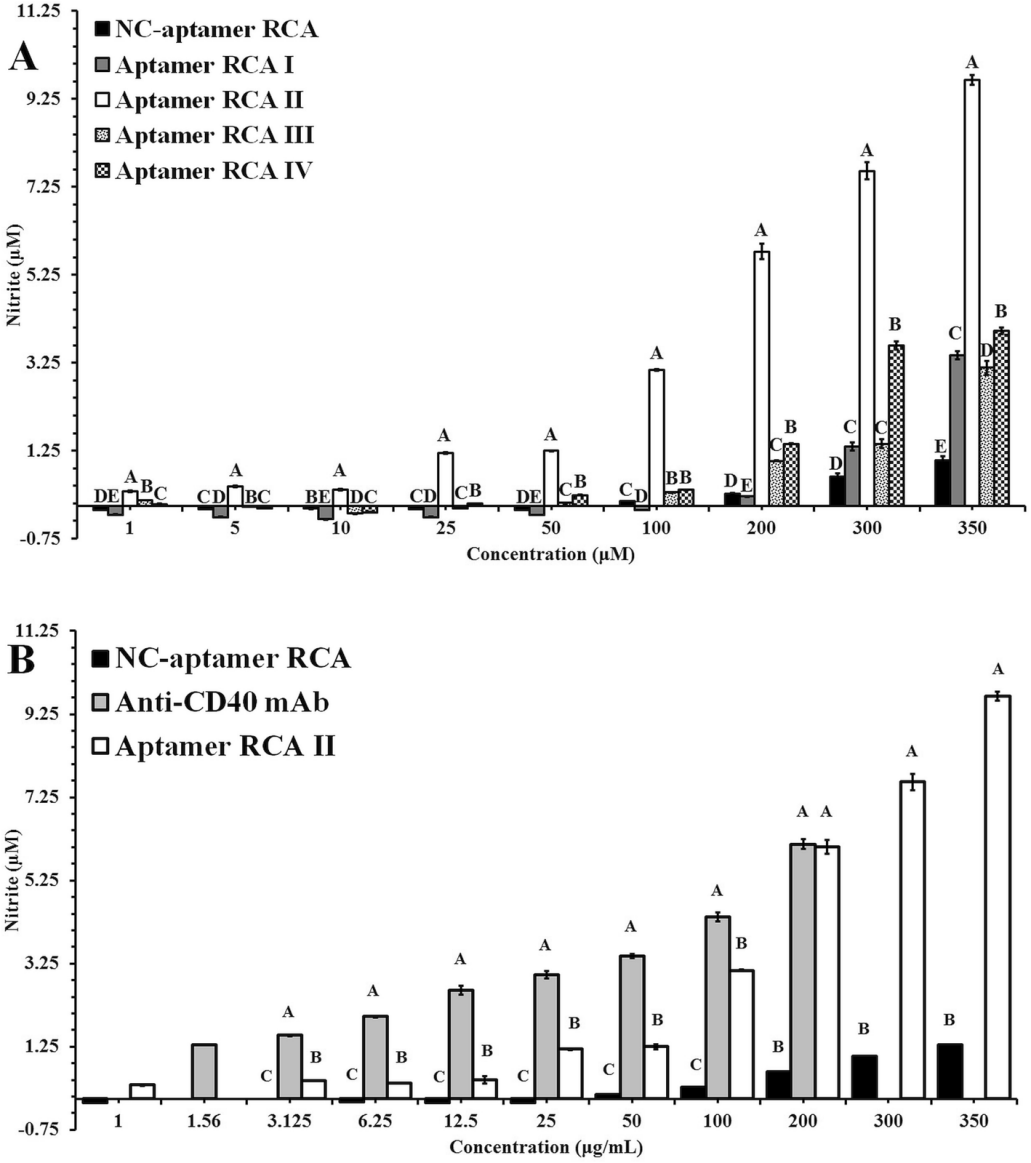
Nitric oxide liberation from chicken HD11 macrophage cell line over increased concentrations of RCA products ((**A**) RCA I to RCA IV in comparison with NC-RCA, (**B**) RCA I in comparison with NC-RCA and anti-chicken CD40 MAb). The RCA products were incubated overnight with the HD11 macrophage cell line in concentrations 1, 5, 10, 25, 50, 100, 200, 300 and 350 (μg/mL) in triplicates. The nitric oxide liberated was measured using Griess assay and showed slop increasing in regard to dose applied. The result was compared to anti-chicken CD40 mAb which is known to have the effect on this cell line (Chen et al., 2010) and negative control RCA (two candidates randomly picked from the library and was not enriched in the Illumina sequencing results). Multiple way ANOVA and Tukey honest significant difference test (HSD) were applied to detect significant differences within treatments. Different uppercase letters represent difference at *p* < 0.0001.

The aptamer RCAs that were stabilized by addition of the Spacer-complement were used to treat the chicken HD11 macrophage cell line^30^. Briefly, the 4 templates (Supplementary Table S1) were circularized by ligation, which was then used to produce 4 aptamer RCAs, namely Aptamer RCA I to Aptamer RCA IV. The results of Griess assay showed that there was elevation in nitric oxide (NO) liberation in the media after incubation with each of all 4 aptamer RCAs. However, there was significant variability in the efficacy and potency among the aptamer RCAs (Fig. 2A). The response was dose-dependent and Aptamer RCA II demonstrated significantly higher responses as compared to all other groups for all concentrations evaluated (Fig. 2A). On the contrary, the result from the aptamer RCA which consists of two DNA sequences from the aptamer library that were not enriched according to the Illumina sequencing result (Negative control) showed no activation of the cell line. Aptamer RCA II was also compared with the anti-chicken CD40 mAb (mAb 2C5) that was previously shown to stimulate the chicken HD11 cells^19^ as the positive control (Fig. 2B). The response of the chicken HD11 cell was also measured by adding lipopolysaccharide (LPS) as the general positive controls for this assay (data now shown). The stimulation of chicken macrophage HD11 cells with Aptamer RCA II was in fact comparable with that of the positive control (mAb 2C5)^30,34,35^.

### MALDI-TOF mass spectrometry and LC–ESI–MS

**Figure 3:**
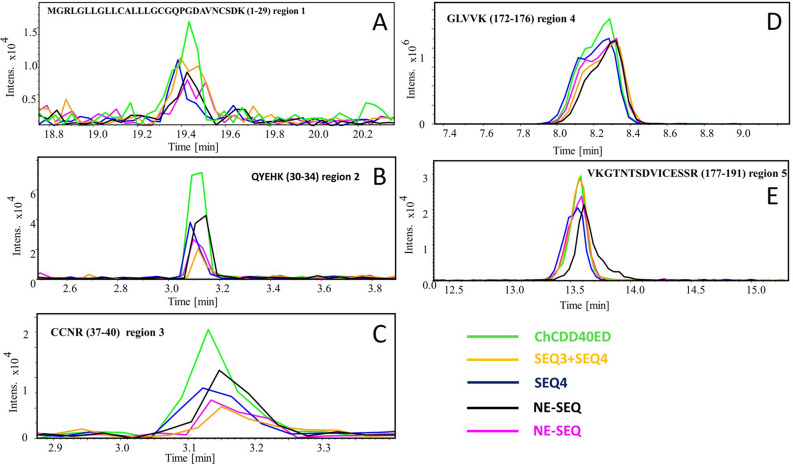
LC–ESI–MS results. After the incubation of the chCD40ED protein (25,712 Dalton) with the aptamers sequences (SEQ3, SEQ4, SEQ3 + SEQ4 or NC-SEQ), the protein was subjected to the action of trypsin digestion for overnight. Three distinct regions (Regions 1, Region 2 and 3 in (**A**), (**B**) and (**C**), respectively; *see* also Fig. 4B) on the protein sequence were protected from the digestion with trypsin enzymes (**A**,**B** and **C**), mostly by SEQ3 + SEQ4. The data collected at different time point (s) were plotted to show the intensity of the peaks. Other regions in Figure (**C**), (**D**) where little protection is provided by aptamers are also shown as a negative control.

**Figure 4:**
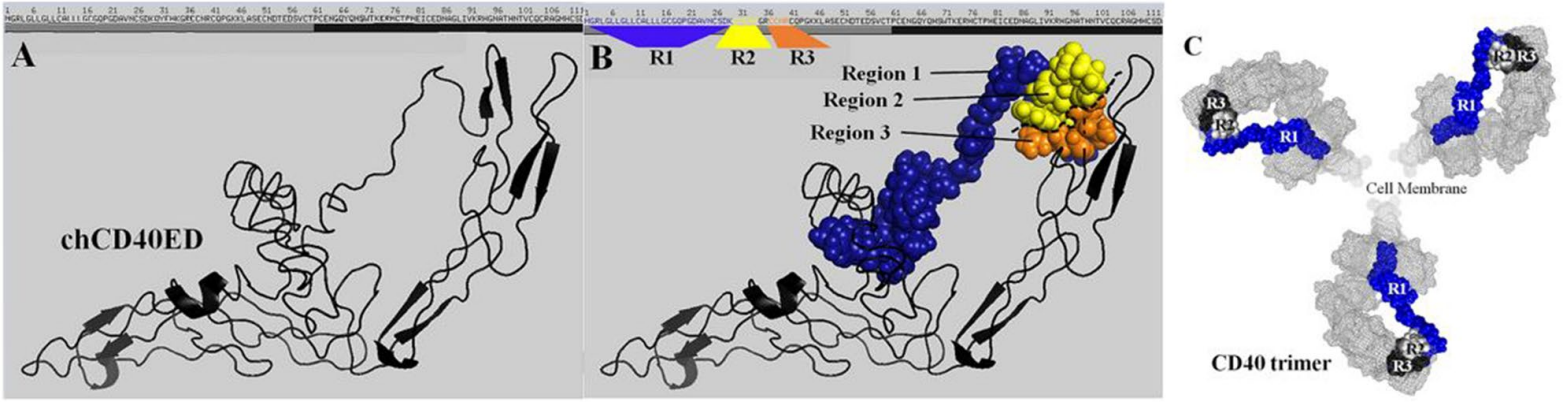
Virtual tertiary structure of chCD40ED showing the potential aptamers linking sites. PyMOL 2.2 software (https://pymol.org/2/) was used for modeling and visualization. (**A**) Cartoon modeling shows the tertiary structure of the protein. (**B**) Space-filling model representing the virtual aptamers-protein interaction sites. Three regions (blue, yellow and orange) determined by MALD-TOF–MS experiment. The blue region represents long *flabby* peptide sequence where CD40L attach. (**C**) Top view of the 3D structure of the chCD40ED. This model shows virtual trimer configuration of CD40 receptor (Mesh model, gray) with the potential aptamer linking sites (space-filling model, blue, white and black). These sites located in the inner of the receptor resembling the actual ligand binding sites (see below).

The MALDI-TOF–MS results showed that the MW of this protein was 25,712 Dalton (data not shown)^36^. LC–ESI–MS was used to discover the binding sites of CD40 protected by DNA aptamers SEQ3 and/or SEQ4. Trypsin is a serine protease and normally active at physiological conditions catalyzing hydrolysis of proteins at lysine and arginine residues. Limited proteolysis experiments at physiological conditions coupled with LC–ESI–MS analysis can reveal solvent accessible surface with lysine and arginine residues. When the CD40 forms a complex with DNA aptamers, some of these solvent bound lysine and arginine residues become less accessible by Trypsin. Hence, LC–ESI–MS intensities of tryptic peptides with these lysine and arginine residues are expected to be lower in signal intensity as compared to the control chCD40ED without the aptamers. The results of extraction ion chromatograms corresponding to tryptic peptides for control protein without aptamer (green), SEQ3 (pink), SEQ4 (blue), SEQ3 + SEQ4 (yellow), and NC-SEQ (black), shown in Fig. 3A–C suggest the binding of the aptamers to CD40 blocked Trypsin digestion of 1–29 (Region 1) 30–34 (Region 2) and 37–40 (Region 3) at N-terminus of CD40. Also, Fig. 3D–E shows the regions (covering 172–191) that resulted in almost no change in the extracted ion chromatogram intensities as compared to the protein only control. In support of the result of the Griess assay where the NC-aptamer RCA was able to stimulate NO liberation at low level (Fig. 2), the NC-aptamer also showed low level, yet detectable protection activity against the action of trypsin enzyme. However, SEQ3 and SEQ4 showed greater and more consistent protection in Regions 2 and 3 as compared to NC-SEQ (Fig. 3B,C, respectively). On the contrary, the result in other two regions show evidence of little protection (Fig. 3D–E), indicating no binding of the aptamers. In addition, to determine the site(s) where the aptamers bind the target protein, the data from the LC–ESI–MS were subjected to virtual protein tertiary structure analysis (Phyre2 Protein Fold software: http://www.sbg.bio.ic.ac.uk/~phyre2/html/page.cgi?id=index). According to this analysis, SEQ3 and SEQ4 interact with the target protein at the N-terminal (Fig. 4B) and the addition of the two aptamers together acted synergistically (Fig. 3B,C). However, the actual sites where these aptamers interact with the protein were not investigated further in this study. In the process of building the tertiary structure of our target protein, these significant peptide regions have not been shown by all the software we tested. This could be attributed to the fact that these regions are flabby, and could not be predicted reliably. Interestingly the three predicted aptamers-protein binding regions are included in these predicted flabby regions (Fig. 4A). It is important to note that in the actual CD40-CD40L interaction, the ligand is spanning the inner parts of the receptor, pulling the extra domains inward leading to oligomerization of the receptor^37^. In our receptor-aptamer interaction model, the receptor is bound apically by Aptamer RCA II. Clustering of CD40 receptor trimers at the solvent-exposed flexible regions of the aptamer appears to be a critical step that causes the same effect as the receptor-ligand clustering and oligomerization (Fig. 4C).

### Evaluation of the novel immunization complex in the chicken model

**Figure 5:**
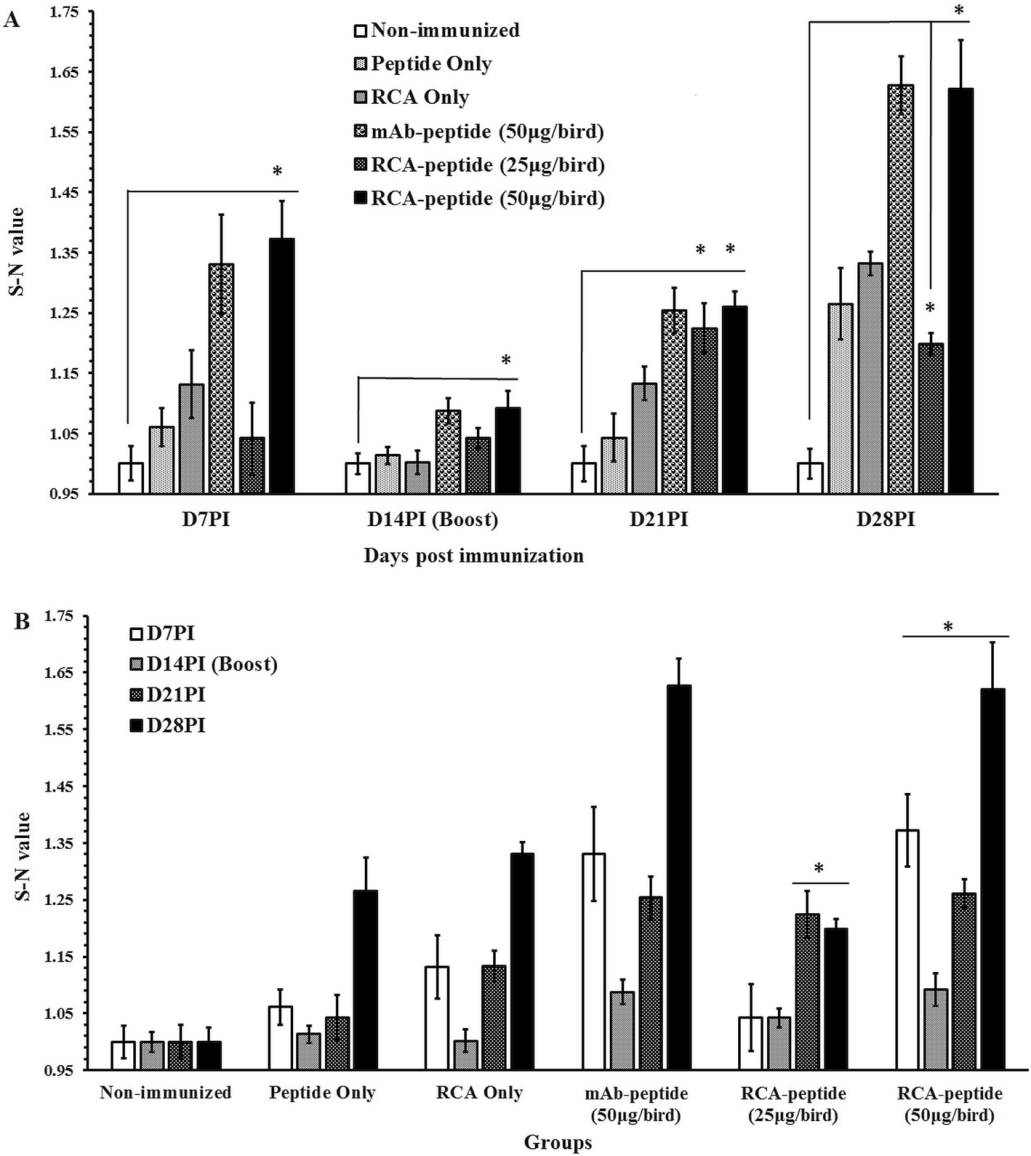
Normalized ELISA results representing S–N values of the anti-M2e IgG in chicken sera at weekly intervals after immunization. Birds (n = 20) were immunized at day 7 and day 21 via s/c route with (1) None (negative control), (2) M2e peptide, (3) aptamer RCA II, (4) mAb-SA-M2e, (5) aptamer RCA II-SA-M2e complex at 25 μg/bird or 50 μg/bird. The normalization calculations S–N = (any read—mean reads of the non-immunized group). The mean of each group calculated accordingly. Results show mean ± SE and the differences are according to ANOVA and student *t*-test. Asterisks represent statistical differences (*p = 0.0001) from the non-immunized group.

As discussed earlier, natural or artificial activation of the CD40 receptor could lead to many desirable effects over the course of the immune response. When B cell is involved in CD40-CD40L crosslinking, high antibody titer, antibody isotype-switching and affinity maturation are obvious^38,39^. In this study, we wanted to test the ability of aptamer RCA products indirectly conjugated with hapten peptide to act as an adjuvant directed toward the CD40 receptor. For this purpose, we chose Aptamer RCA II, because this one induced the most robust activation of HD11 macrophage cell line. We evaluated the ability of the immune complex (aptamer RCA-SA-M2e) in which Aptamer RCA and M2e are conjugated together through Streptavidin (SA) (Supplementary Figure S5) to elicit M2e-specific immune response using two different doses in comparison with several negative control groups (No-vaccination group, SA-M2e, and aptamer SA-RCA) and with our previous immune complex using mAb 2C5 (mAb-SA-M2e) as a positive control (Supplementary Table S4). As reported previously, the chickens immunized with mAb 2C5 conjugated with M2e peptide could induce high level of antibody titer directed against M2e peptide^19^. Our results showed that the anti-M2e IgG antibody titer was detected as early as 7 days post immunization and particularly aptamer RCA II-SA-M2e complex at high dose showed significantly higher response as compared to No-immunization control (Fig. 5A). This titer remained significantly high to the end of the experiment especially for birds with the high dose. These results are comparable with that obtained with mAb-SA-M2e. The administration of 50 μg/bird of aptamer RCA II-SA-M2e complex induced significant level of anti-M2e IgG titer for a long-term period (*p* = 0.0001) (Fig. 5B).

The same immune complex at lower dose (25 μg/bird) failed to induce significant level of anti-M2e IgG in the sera at 7- or 14-days post first immunization. However, when this immune complex was used for boosting at day 14, it induced significant level of the antibody titer at 28 days post the first immunization (Fig. 5). All birds showed retarded immune response at day 14 post immunization. Our record showed some management issues during the day before the onset of blood sampling. This might have cause stress in the birds at that particular time point. However, it appears that the birds have compensated the immune response against the immunogen in later weeks.

## Conclusions and significance

In recent years, aptamers and aptamer-based molecules have been used in place of mAbs in many areas of molecular biology and biotechnology. However, expansion in this field is yet to overcome the limitations of the use of the aptamers due to poorly understood molecular properties of these molecules. In this study, we demonstrated that aptamer RCAs are capable of activating chicken macrophage HD11 cell line via interaction with the CD40 receptor. We report the first description of the use of aptamer RCAs as an agonist for a receptor for the purpose of enhancing immune response, targeting chCD40ED. The synthetic aptamers in the form of aptamer RCA mimic the naturally occurring receptor/ligand interaction, leading to receptor oligomerization. We also characterized the aptamer binding sites on chCD40ED by demonstrating that DNA aptamers can block the action of the trypsin enzyme on the target protein by using LC–ESI–MS experiment. Herein, we determined the peptide regions where the aptamers bind to the protein. In this study, we used monomer chCD40ED instead of the trimer as our target protein for aptamers selection. However, our protein was expressed in eukaryotic cell line system, which should support the post-translation modification. In naturally occurring CD40-CD40L interaction stoichiometry, upon initial physical contact of the ligand with its cognate receptor, the receptor undergoes clustering at the extracellular domain, and signal transduction occurs through the cytoplasmic domain^40^. The receptor’s extracellular crosslinking is what leads to receptor oligomerization and this observation was recorded with the natural and artificial agonists of the CD40; i.e., CD40L and anti-chicken CD40 mAb^40,41^. The ligand has to interact with the receptor in a specific manner to induce the response. Interestingly, soluble CD40L was not able to induce the expected receptor signaling nor many other anti-chicken CD40 mAbs. The results suggest that there are specific patterns and sites for ligand binding to allow efficient signal transduction^42^. Our aptamers did not show any agonistic activity when administered to the macrophage cell line as an individual nor combined aptamers (data not shown). The stimulation only occurred with aptamer RCA. Similarly, we have proven that not all the aptamers we have selected were able to significantly oligomerize the receptor; only those that were able to link specific receptor sites were successful^43–45^.

Our goal was to create synthetic molecule that could be preferably beneficial when loaded with appropriate antigen to act as an adjuvant. The flexibility we provide with the use of aptamer-based RCA makes it easier to introduce the aptamers into the biological systems. Indeed, the aptamer RCAs are naturally protected against any nuclease enzyme activity at the downstream due to the attached circular template (data not shown).

In the chicken study, despite the use of relatively low concentration of the hapten, our Aptamer RCA II-SA-M2e complex successfully induced high level of anti-M2e IgG antibody titer in the chicken sera at the dose of 50 μg/bird. With this dose, the elevation of the antibody titer started to increase as early as seven days post immunization and remained high to the end of the experiment. The action of this immune complex proves that this complex is with high potency and efficacy. These results are comparable to the result with mAb-SA-M2e complex. However, lower dose of the same complex (25 μg/bird) yielded only low level of the antibody titer and needed a booster dose to sustain relatively acceptable level of the same antibody titer at the end of the experiment.

Our immune adjuvant might work as an universal adjuvant with any short soluble antigenic peptide. The immune response course with this DNA aptamers-based RCA immunogenic adjuvant would pass through APCs-T_H_ cells interaction pathway.

Finally, targeting the CD40 receptor with DNA aptamers as cheaper molecules, easier to prepare and more stable, would improve the effectiveness of many vaccines not only in the field of animal husbandry but also in the development of human vaccines in general.

## Methods

### Study approval

All procedures for handling animals in this study were approved by the Institutional Animal Care and Use Committee (IACUC) of the University of Arkansas. All methods were carried out in accordance with relevant guidelines and regulations.

### Preparation of the target protein

Our target protein was the chicken CD40 receptor; however, the recombinant protein used for the aptamer selection was chicken CD40 extracellular domain. The chCD40ED protein was expressed in HEK293 Freestyle cells in protein-free medium and purified by Antagene (Santa Clara, CA) at the concentration of 1 mg/mL. According to the mass-spectrometry analysis, the protein was with MW around 25,712 Da. The protein stock was diluted with sterile 1 × phosphate buffer saline (1 × PBS, w/v) to the concentration of 15 μg/L and aliquoted into 1 ml in sterile tubes and stored at − 80 °C.

### Single stranded DNA library and primers

A randomized ssDNA library, forward and reverse primers were purchased from Integrated DNA Technologies (IDT, Coralville, IA). The ssDNA library was flanked with 20 nucleotides (nt) forward primer sequence at the upstream and 20 nt reverse primer binding site at the downstream. The randomized region of the library was with 40 nt with theoretical diversity of 1 × 10^24^ random sequences (Supplementary Table S1). The random region of the library consists of randomly incorporated bases of A, T, G and C at equal ratio at each position. The reverse primer was phosphorylated at 5′ end to allow the action of the λ exonuclease enzyme in the later step during the enrichment cycles.

### In vitro selection procedure

The procedure of aptamer selection was as described elsewhere^20,21^. At the first round, 35 μl of the ssDNA library (1 μg/μl) was denatured by heating to 95 °C for 10 min, cooled down to 4 °C for 5 min and incubated at room temperature (RT) for 10 min. The whole procedure, buffers and timing were as previously described^46,47^. Each following SELEX enrichment round used only 75% of the volume of the target protein (using the stock solution at the same concentration) in the previous round.

### PCR and dsDNA digestion into ssDNA

After each enrichment cycle, the resulting ssDNA was amplified via 25 cycles of PCR amplification. The PCR was conducted using G2 Hot Start Green Master Mix (Promega). The reaction mix consisted of 1 μl (10 μM) from the of the DNA library (enriched aptamers in the later cycles) as the template, 2 μl of each forward and phosphorylated reverse primers (4 μM), and 12.5 μl of 2 × mater mix (pH 8.5). Nuclease-free water was added to adjust the final reaction volume to 25 μl. The thermal cycles were composed of 95 °C for 5 min as the denaturing step, followed by 25 thermal cycles of 95 °C for 30 sec, 56 °C for 15 sec and 72 °C for 15 sec. The final extension was at 72 °C for 5 min. The PCR products were monitored on 1% agarose gel stained with SYBR Safe dye (Thermo Fisher Scientific). The PCR-resulted double-stranded DNA (dsDNA) was subjected to digestion with λ exonuclease enzyme (NEB) to convert dsDNA into ssDNA to be used for the next round (Supplementary Table S1). The digestion reaction conditions were as described by the manufacturer.

### Sanger sequencing

After 6 rounds of selection, the PCR product was cloned and sequenced using Sanger sequencing method. The cloning process utilized the pGEM-T and pGEM-T easy vector systems (Promega). As instructed by the manufacturer, the PCR product was inserted into the kit vector in ligation buffer. The insert-containing plasmid DNA was transformed into electrocompetent TOP10 *E. coli* (Invitrogen, Carlsbad, CA, USA) by pulsed high voltage electroporation (1750 V for 5 ms) (ECM 830 electroporation generator, Fisher Scientific). After the transformation, 500 μL of SOC media (Invitrogen, Carlsbad, CA) was added and the bacteria was incubated for 2 h at 37 °C with shaking. The SOC medium-containing bacteria was streaked on Luria–Bertani medium with ampicillin (100 μl/plate) (ThermoFisher Scientific)/ IPTG (Calbiochem, San Diego, CA)/5-bromo- 4-chloro-3-indolyl-β-D-galactoside [X-Gal]. After overnight incubation, the culture was screened and 15 white colony candidates were harvested. The identification of positively cloned colonies depends on the color differentiation. Bacteria do not harbor the pUC18 plasmid will be killed by the ampicillin, bacteria do not carry the insert will appear in blue color colonies because of the X-Gal, and the bacteria that harbor the insert will appear in white colonies. One extra blue colony was included as a positive control for the assay. Those chosen cells were lysed and the DNA was purified using QIAprep Miniprep kit (Qiagen, Hilden, Germany) and sent for Sanger sequencing (Eurofins, Louisville, KY).

### Illumina sequencing

The procedure of high throughput Illumina sequencing was carried out as previously described^46^. Resulting sequences were analyzed using “Galaxy Bioinformatics”, a free software at https://usegalaxy.org/ and “FastQC” version 0.11.5, a free software at www.bioinformatics.babraham.ac.uk^49–51^. Samples from rounds 1, 6, 8 and 10 were prepared for Illumina sequencing. Each selected round’s ssDNA was barcode-tagged and used as a template to be amplified by PCR using Illumina sequencing primers (Supplementary Table S1). Twenty-five PCR cycles have been applied using KOD hot start DNA polymerase and buffer (Sigma-Aldrich, CA). All PCR products were sent for sequencing at the University of Wisconsin Biotechnology Center (UWBC, WI).

Total number of the reads by the Illumina sequencing were ~ 16 million reads; however, 5% of the total reads were neglected due to their improper length or quality. The rest of the reads were tracked according to their barcodes. After analyzing the results, the highly-yielded aptamers were tested for the free energy change (ΔG[kcal.mole^−1^]) of the three dimensional structure using the OligoAnalyzer 3.1 Tool at Integrated DNA technologies website (www.idtdna.com) and the most reliable ones have been chosen to be tested by the next step.

### Dot-blot hybridization assay

Dot-blot assay was applied to detect the affinity of the candidate aptamers to the target protein. The assay was as described elsewhere^46,47^. According to the Sanger sequence and the Illumina sequencing results, the candidate aptamers were ordered as biotinylated at the upstream (IDT, Coralville, IA). Immune-Blot PVDF membrane (0.2 μm, Bio Rad Laboratories, CA) cut into 8 strips of 6 cm^2^ (1 cm^2^/trt). 10 μl (15 μg/mL) of the target protein was dropped onto the center of 5 of the test squares. After drying, the stripes blocked with KPL blocking buffer for 30 min and washed (3X) with 1X KPL washing buffer (KPL, Gaithersburg, MD). Concentrations of 12, 25 and 50 μM of the aptamers were dropped onto the center of the test squares on the membranes. The membranes sat to air dry and washed (3X) with 1X KPL washing buffer. Streptavidin-conjugated alkaline phosphatase (KPL, Gaithersburg, MD) (diluted 1:500) poured on the membrane for 30 min and another course of washing followed. Finally, the color developed by adding 5-bromo-4-chloro-3-indoxyl-phosphate and nitro blue tetrazolium (BCIP/NBT) substrate (KPL, Gaithersburg, MD). The reaction stopped by pouring tap water thoroughly.

### Rolling circle amplification

According to the Dot-blot assay, the chosen aptamers were with good affinity to the target protein, yet, their ability to sustain the oligomerization of the CD40 receptor in chicken HD11 macrophage cell line was questionable. To achieve the highest possibility for this process, rolling circle amplification (RCA)-containing polyvalent aptamers was suggested. All the eight candidate aptamers were grouped into four groups with two aptamers each to design four complementary templates for RCA technique. The process of coupling two aptamers in one template started as applying the ones with the highest percent of exponentially enriched aptamers sequences according to the Illumina sequencing, the following template design annealing procedure. The ligation reaction conditions were as recommended by the manufacturer.

For the polymerization, ɸ 29 DNA polymerase enzyme (NEB) was used. All the reaction conditions were as recommended by the manufacturer except that the reaction was hold for 16 h and then heat-inactivated at 65 °C for 10 min. After each enzymatic treatment, the DNA was purified by ethanol precipitation. For the addition of Spacer-complement, the RCA quality and quantity were evaluated using microplate reader (BioTeck). Spacer-complement was mixed with Aptamer RCA II at the molecular ratio of 2:1. Again, the Aptamer-Spacer-complement mixture was hold on heating for 10 min at 95 °C and for 50 °C for 5 min and then ice-cooled immediately to apply the annealing status.

### Measuring activation of chicken HD11 macrophage cell line

Chicken HD11 macrophage cell line was propagated on 96-well plate at 37 °C in a humidified atmosphere having 5% CO_2_ in Dulbecco’s modification of eagle’s medium (DMEM) (Mediatech, Manassas, VA). The medium enriched with 8% fetal bovine serum (Atlanta Biologicals, Lawrenceville, GA) and 5% chicken serum (Sigma, St. Louis, MO). All treatments were applied when the confluence of the batch was 80%. After the treatment, the plate was incubated at the same conditions for extra four hours. When the time passed, the contents of every well was collected, centrifuged and the supernatant was subjected to Griess assay to detect the nitric oxide liberation in the medium. All cell line work was carried out in Dr. Berghman’s laboratory, Texas A&M.

### MALDI-TOF–MS and LC–MS mass-spectrometry

First, both protein and individual ssDNA aptamers were subjected to matrix-assisted laser desorption ionization time of flight mass spectrometry (MALDI-TOF–MS) using Bruker Daltonics, MALDI reflex III time of flight instrument to confirm the expected molecular weight for both protein and the aptamers. Then, the protein was subjected to full trypsin digestion, followed by liquid chromatography tandem mass spectrometry (LC–ESI–MS/MS) using Agilent 1200 series micro flow HPLC in line with Bruker Amazon-SL quadrupole ion trap ESI mass spectrometer (QIT-ESI–MS) to confirm the protein identity as the CD40 ligand from *Gallus gallus* using standard method described in elsewhere^52^. To have fully understood aptamers-protein binding process, limited proteolysis liquid chromatography electrospray ionization mass spectrometry (LC–ESI–MS) experiment has been conducted using Bruker ESQUIR-LC quadrupole ion trap (QIT) without MS/MS and intensities of the extracted ion chromatograms corresponding each tryptic peptide were used to determine the relative inhibition for the enzyme activity toward the CD40 protein due to aptamers binding. LC–ESI–MS was operated using standard method protocols described elsewhere^52,53^ (ref https://academic.oup.com/ps/article/88/2/372/1563011). One microgram of the chCD40ED protein was subjected to the digestion with 20 ng of trypsin (Promega) containing 25 mM NH_4_HCO_3_ ~ 7.5 pH buffer at 37 °C for 1 h with and without the candidate aptamers. In addition, negative control ssDNA aptamers random sequence was included***.*** After the incubation period passed, the enzyme action was chemically inactivated by adding 5% FA in 60% acetonitrile (ACN) and the resulted digest was subjected to LC-ES-MS.

### Birds, random mixing and management procedure

Total 120 broiler one-day-old chicks were tag-divided into six groups (n = 20) in comingle pen. The chicks were single gender Cobb-Vantress off-sex obtained on the day-of-hatch from the Fayetteville hatchery, Fayetteville, AR. The chicks were mechanically block randomized by moving 1/3 of each of the chicks from three boxes to a single new chick box, thus equally re-mixing each of the boxes. This was done in case boxes were derived from different hatchers and/or potentially different breeder sources (this variability has historically been the most critical for randomization effects). The management practice took the consideration of the broiler management guide of Cobb (https://www.cobb-vantress.com/).

### Aptamers, rolling circle amplification products and M2e peptide

Aptamers and RCA products were prepared as described earlier. However, aptamer RCA products were produced with a biotinylated primer (IDT, Coralville, IA) to facilitate conjugation with Streptavidin in the later step.

### Peptide immunogen

A commercially available synthetic amino-terminus biotin labeled M2e peptide was ordered as biotinylated (GenScript Inc. Piscataway, IL). This peptide was with the sequence “NAWSKEYARGFAKTGK” representing the ectodomain of Influenza virus matrix protein 2. Conceptually, two biotinylated RCA molecules and two biotinylated M2e peptide were conjugated by one streptavidin molecule as described in the next section.

### Immunizing complex preparation, doses, timing and administration

Immunization complexes were prepared as described elsewhere^19^, Streptavidin (Thermo Fisher Scientific) was used as the central molecule that conjugate other molecules in the immune complex (Figure S5). The peptide was ordered as biotinylated (GenScript Inc. Piscataway, IL), whereas the biotinylation of Aptamer RCA II was accomplished by using a biotinylated primer for RCA procedure. The biotinylated peptide (0.2 μg) was mixed with the biotinylated Aptamer RCA II (50 μg) in 1 to 1 ratio on molecular basis in 1 × PBS with rotation at room temperature for 3hrs. The M2e-Aptamer RCA II mixture was then added to streptavidin in 2 to 1 ratio and stirred at 4 °C for 3 h. The same procedure was followed when preparing the anti-CD40 monoclonal antibody-based immune complex, the peptide alone or Aptamer RCA II alone. Similarly, the peptide or Aptamer RCA II alone ratio to the streptavidin was 2 to 1. The immunizing doses and timing were as described in Supplementary Table S4. The total volume of the calculated doses for either immunizing formula was 0.4 mL/bird. The excipient of the immunizing complex was emulsified PBS [5% (v/v) squalene, 0.4% (v/v) Tween 80 (Sigma-Aldrich, St. Louis, MO) in PBS].

### Blood sampling

Blood sampling was at weekly intervals in a sterile procedure. The blood were collected from the jugular vein at week 1 and from the wing vein at the next four weeks. Sera were separated from the whole blood by centrifugation, and then stored at − 20 °C.

### Quantification of specific IgG by ELISA

The level of the anti-M2e IgG titer in the sera was quantified by ELISA. All serum samples loaded in duplicates throughout the assay. The ELISA procedure started by incubation of the biotinylated M2e with 5 μg/mL goat anti-biotin antibody (Thermo Fisher Scientific) in 0.05 M carbonate-bicarbonate buffer (pH 9.6) in 1 to 1 ratio for 2hrs at RT with rotation. This M2e-antibody complex then coated a 96-well microtiter plate (MaxSorb, Thermo Fisher Scientific) overnight at 4 °C. Next, the un-absorbed supernatants were removed and the plate was washed with 1xPBS-T and blocked with 100uL commercial blocking buffer (Thermo Fisher Inc.) for 1 h at RT. After a course of washing with 1 × PBS-T, the plate was incubated with the investigated sera [(100μL), diluted 1:500 in PBS to blocking buffer (1:1), 1% normal goat serum (v/v) and 1% normal rabbit serum (v/v)] overnight at 4 °C. After another course of washing, the plate was loaded with 100 μL of horseradish peroxidase-conjugated, affinity purified isotype-specific rabbit anti-chicken IgY antibody (Thermo Fisher Scientific). The dilution of the secondary antibody was 1:30,000 and the diluent was with bovine serum albumin (BSA) and 1% normal goat serum (v/v). The plate was set for 1 h at RT and a course of 5 × washing followed. For developing the color reaction, we used OptEIA TMB substrate (BD, Lakes, NJ). The color developing process was following the manufacturer instructions and the reaction terminated by adding 1 N sulfuric acid (50 μL/well) after 5 min for all the trials. Absorbance was measured by using BioTeK microplate reader (BioTek Instruments, Inc., Winooski, VT) at 450 nm using Gen5 version data analysis software (BioTek Inc).

### Statistical analyses

Statistical analyses of Aptamer RCA II dose response in the first experiment were depending on the Variance as a completely randomized design. Multiway Analysis of Variance (ANOVA) carried out to show variation between treatments, using the General Linear Models procedure of SAS software (JMP Pro 13, free software by the U of A). In all data sets, mean ± standard deviation and p value were the main comparison elements. Tukey HSD was used to determine the significant differences among means at *p* < 0.001.

The relative levels of the anti- peptide antibody in the sera was normalized by calculating the value of each sample to the mean value of the non-immunized group as the negative control (S–N). The average mean of the readings of the non-immunized birds was the baseline to calculate the S–N values of all groups. One-way analysis of variance (ANOVA) with least significant differences (L.S.D.) of the means of the S–N values and student’s *t-*test were used to determine the significant differences between the treatments. All data were analyzed using JMP software (SAS institute Inc., Cary, NC, software by the University of Arkansas). Statistical differences across different groups in the chicken study were considered significant at *p* = 0.0001.

### Ethics approval

The animal study was reviewed and approved by University of Arkansas Institutional Animal Care and Use Committee.

## Supplementary information

Supplementary Information.

## Data Availability

The datasets analyzed during the current study are available from the corresponding author on reasonable request.
