# Supplementary material for: DNA aptamer-based rolling circle amplification product as a novel immunological adjuvant
Source: Sci Rep. 2020 Dec 17;10:22282. doi: 10.1038/s41598-020-79420-w (PMC7747709; doi:10.1038/s41598-020-79420-w)
Supplement: Supplementary file 1 — Supplementary Information. [file 41598_2020_79420_MOESM1_ESM.docx]

**SUPPELEMENTARY MATERIALS**

**DNA aptamer-based rolling circle amplification product as a novel immunological adjuvant**

Adil S. Al-Ogaili, Rohana Liyanage, Jack O. Lay, Jr., Tieshan Jiang, Christine N. Vuong, Shilpi Agrawal, Thallapuranam Krishnasawmy Suresh Kumar, Luc R. Berghman, Billy M. Hargis and Young Min Kwon

**Table S1. DNA sequences of the aptamer library, various oligonucleotides and templates for RCA reactions**

| Description | DNA Sequence (5’🡪3’) |
| --- | --- |
| Library | CCG AAT TCG AAG GAC AAG AG (N) ^†^_40_ TCT TT TAT GCT ACG TCC CGC |
| Forward primer (FP) | CCG AAT TCG AAG GAC AAG AG |
| Reverse Primer (RP) | GCG GGA CGT AGC ATA AAA GA |
| Phosphorylated RP | Phosph/GCG GGA CGT AGC ATA AAA GA |
| Illumina FP | AAT GAT ACG GCG ACC ACC GAG ATC TAC ACT CTT TCC CTA CAC GAC GCT CTT CCG ATC TNN NNN GG(BC)^‡^TT– (FP) |
| Illumina RP | (RP)–TAG AGC ATA CGG CAG AAG ACG AAC |
| Aptamer RCA I template | Phosph/GCA TCT GAA C GCG GGA CGT AGC ATA AAA GAA GGA GAC CCG TAT GCA TAA CGG TGA CAC GAT CAA GTC GAA CTC TTG TCC TTC GAA TTC GG GAT CCA CCG GTA GCA GCG GGA CGT AGC ATA AAA GAG GAA AGT ATT CAA GGT TAA AAT GTA CGT GTG GAC GCG AAT CTC TTG TCC TTC GAA TTC GG GGA ACG TCT T |
| Aptamer RCA II template | Phosph/GCA TCT GAA C GCG GGA CGT AGC ATA AAA GAC GAA TTC ACT GCT CCA TTT ACA CCC CAT TAC CAA TTC CAC CTC TTG TCC TTC GAA TTC GG GAT CCA CCG GTA GCA GCG GGA CGT AGC ATA AAA GAC CTA GCC CTG CTC TTC TGA TCC CTA TTC CAT GTA GCC CTA CTC TTG TCC TTC GAA TTC GG GGA ACG TCT T* |
| Aptamer RCA III template | Phosph/GCA TCT GAA C GCG GGA CGT AGC ATA AAA GAG CGG GAC GTA GCA TAA AAG AAA ACC CAA ACT GCA CTT ACT CTT GTC CTT CGA ATT CGG GAT CCA CCG GTA GCA GCG GGA CGT AGC ATA AAA GAG AAC AAT CGG AAC TGG TCA TCC TAG GAC GTC GTG ACT TAC CTC TTG TCC TTC GAA TTC GG GGA ACG TCT T |
| Aptamer RCA IV template | Phosph/GCA TCT GAA C GCG GGA CGT AGC ATA AAA GAC AGA TGA CCA ACC ATA CTG ACT TGA CAG CAT CCC TCA TCT CTC TTG TCC TTC GAA TTC GG GAT CCA CCG GTA GCA GCG GGA CGT AGC ATA AAA GAA TGG GGG TAG TTGC AAA GGA TAG GGG TAA AAG AAA TGT TGC TCT TGT CCT TCG AAT TCG G GGA ACG TCT T |
| NC-aptamer RCA template | Phosph/GCA TCT GAA C ATG GGC AGT TCA AAC TAC GAC ATA GCA GAC CAA GGT ATA G GAT CCA CCG GTA GCA TGA TGC AGG TCC TGT AAT TGG ACT GAT CAC ATA CCT AGA T GGA ACG TCT T |
| RCA primer | GTT CAG ATG CAA GAC GTT CC |
| Spacer-complement (SC) | GAT CCA CCG GTA GCA |

^†^ Random sequence

^‡^ Barcode sequence

*Single underline represents the primer-binding site and the double underline represents the spacer region.

**Table S2. Selected aptamers**.

| Description | Aptamers | Aptamer RCA |
| --- | --- | --- |
| SEQ1  (Sanger) | **CCGAATTCGAAGGACAAGAGCAGACAAACGAGTATTAGGACAGAACGGTAGCACGACTCATCTTTTATGCTACGTCCCGC** | Aptamer RCA I |
| SEQ2  (Sanger) | **CCGAATTCGAAGGACAAGAGAGAAAAAACGAATCCCAGGACAGACCGGCGTGTCGAGTCCTCTTTTATGCTACGTCCCGC** |  |
| SEQ3  (Illumina) | **CCGAATTCGAAGGACAAGAGGTGGAATTGGTAATGGGGTGTAAATGGAGCAGTGAATTCGTCTTTTATGCTACGTCCCGC** | Aptamer RCA II |
| SEQ4  (Illumina) | **CCGAATTCGAAGGACAAGAGTAGGGCTACATGGAATAGGGATCAGAAGAGCAGGGCTAGGTCTTTTATGCTACGTCCCGC** |  |
| SEQ5  (Illumina) | **CCGAATTCGAAGGACAAGAGTAAGTGCAGTTTGGGTTTTCTTTTATGCTACGTCCCGCTCTTTTATGCTACGTCCCGC** | Aptamer RCA III |
| SEQ6  (Illumina) | **CCGAATTCGAAGGACAAGAGGTAAGTCACGACGTCCTAGGATGACCAGTTCCGATTGTTCTCTTTTATGCTACGTCCCGC** |  |
| SEQ7  (Illumina) | **CCGAATTCGAAGGACAAGAGAGATGAGGGATGCTGTCAAGTCAGTATGGTTGGTCATCTCTCTTTTATGCTACGTCCCGC** | Aptamer RCA IV |
| SEQ8  (Illumina) | **CCGAATTCGAAGGACAAGAGCAACATTTCTTTTACCCCTATCCTTTGCAACTACCCCCATTCTTTTATGCTACGTCCCGC** |  |

**Table S3. Aptamers proportion and relative frequency at each enrichment SELEX round**. Sequences of each rounds marked with certain barcode to ensure accurate splitting with the proper software. The proportion of six highly enriched aptamer sequence/round and their relative frequency upon each round have been estimated.

| Round | Barcode | No. of Sequences/round | Sequence | Fraction in total sequence reads | % relative frequency within SEQ3-8 |
| --- | --- | --- | --- | --- | --- |
| 1 | TAGATCGC | 4.438 x10^6^ | SEQ3 | 0.00006 | 2.80 |
|  |  |  | SEQ4 | 0.00011 | 4.67 |
|  |  |  | SEQ5 | 0.00155 | 64.49 |
|  |  |  | SEQ6 | 0.00048 | 19.62 |
|  |  |  | SEQ7 | 0.00018 | 7.50 |
|  |  |  | SEQ8 | 0.00002 | 0.95 |
|  |  |  | SEQ3 | 0.00231 | 9.45 |
|  |  |  | SEQ4 | 0.00198 | 8.09 |
| 2 | CTCTCTAT | 3.9 x 10^6^ | SEQ5 | 0.01408 | 57.66 |
|  |  |  | SEQ6 | 0.00408 | 16.70 |
|  |  |  | SEQ7 | 0.00139 | 5.68 |
|  |  |  | SEQ8 | 0.00059 | 2.43 |
| 6 | TATCCTCT | 4.368 x 10^6^ | SEQ3 | 0.00104 | 4.42 |
|  |  |  | SEQ4 | 0.00133 | 5.69 |
|  |  |  | SEQ5 | 0.00319 | 13.64 |
|  |  |  | SEQ6 | 0.00591 | 25.31 |
|  |  |  | SEQ7 | 0.00053 | 2.26 |
|  |  |  | SEQ8 | 0.01136 | 48.67 |
| 8 | AGAGTAGA | 4.038 x10^6^ | SEQ3 | 0.02725 | 5.30 |
|  |  |  | SEQ4 | 0.07678 | 14.89 |
|  |  |  | SEQ5 | 0.21052 | 40.83 |
|  |  |  | SEQ6 | 0.05697 | 11.04 |
|  |  |  | SEQ7 | 0.04211 | 8.17 |
|  |  |  | SEQ8 | 0.10194 | 19.77 |
| 10 | ACTGCATA | 2.739 x10^6^ | SEQ3 | 0.16631 | 5.29 |
|  |  |  | SEQ4 | 0.31121 | 6.52 |
|  |  |  | SEQ5 | 3.20593 | 67.12 |
|  |  |  | SEQ6 | 0.66189 | 13.85 |
|  |  |  | SEQ7 | 0.17813 | 3.74 |
|  |  |  | SEQ8 | 0.16631 | 3.49 |

**Table S4**. Description of the *in vivo* experimental design. Six groups (n=20) were raised in a comingle pen and immunized with two different doses of RCA-SA-M2e immune complex (i.e. low: 25μg/bird and high: 50μg/bird) for two times. This immune complex composition was compared with anti-chicken CD40 monoclonal antibody-based immune complex that has been given at (50μg/bird) for two times as well. In addition, the tested immune complex was compared with three negative control groups.

| **Group** | **Vaccine** | **Description of the vaccine^⸸^** | **Dose/bird^*^** | **prime/route** | **boost/route** |
| --- | --- | --- | --- | --- | --- |
| 1 | Negative-negative control | No vaccination | NA | NA | NA |
| 2 | Negative control | SA-M2e | 0.2μg | D7/SC | D21/SC |
| 3 | Negative control | SA-aptamer RCA II | 50μg | D7/SC | D21/SC |
| 4 | mAb-based vaccine | 2C5-SA-M2e | 50μg | D7/SC | D21/SC |
| 5 | Aptamer RCA-based vaccine | aptamer RCA II-SA-M2e | 25μg | D7/SC | D21/SC |
| 6 | Aptamer RCA-based vaccine | aptamer RCA II-SA-M2e | 50μg | D7/SC | D21/SC |

1. SEQ1 (ΔG = -1.24)


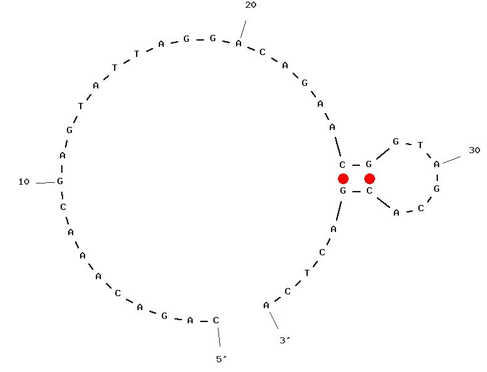


1. SEQ2 (ΔG = -2.78)


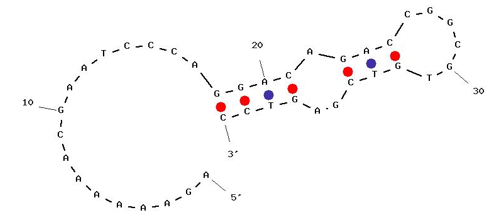


1. SEQ3 (ΔG = 0.70)


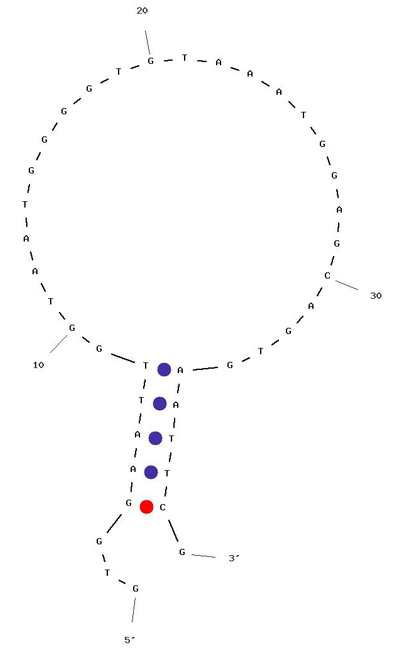


1. SEQ4 (ΔG = -0.71)


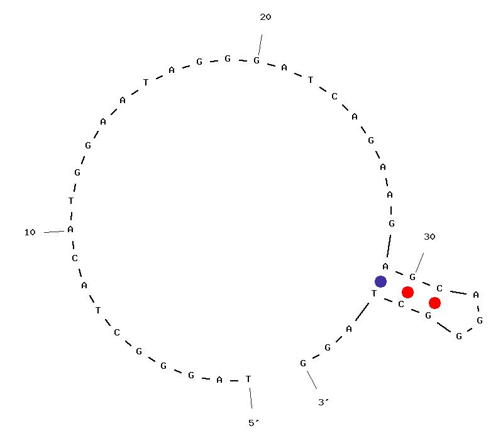


1. SEQ5 (ΔG = -0.72)


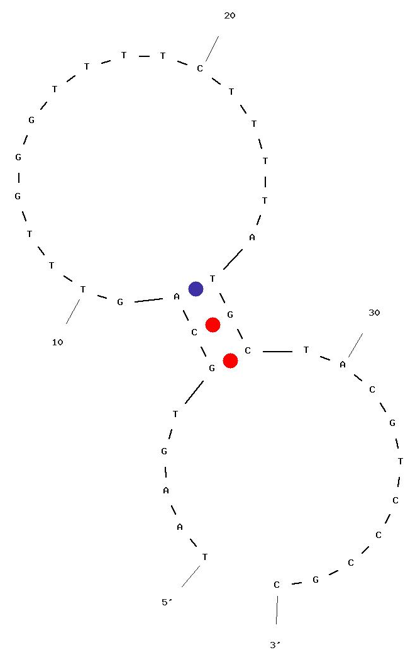


1. SEQ6 (ΔG = -1.03)


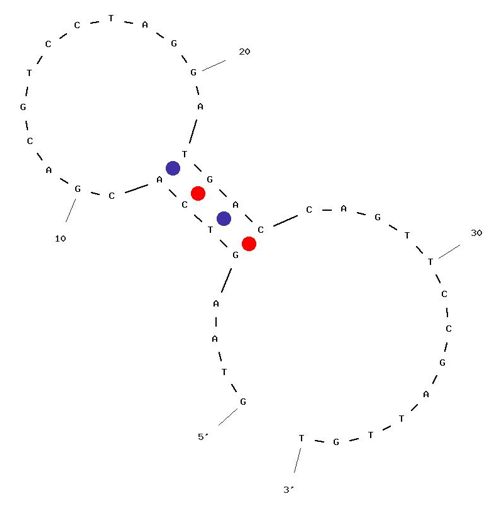


1. SEQ7 (ΔG = -1.04)


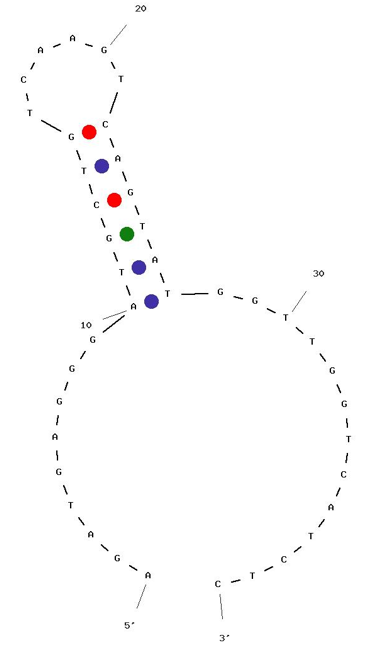


1. SEQ8 (ΔG = -1.66)


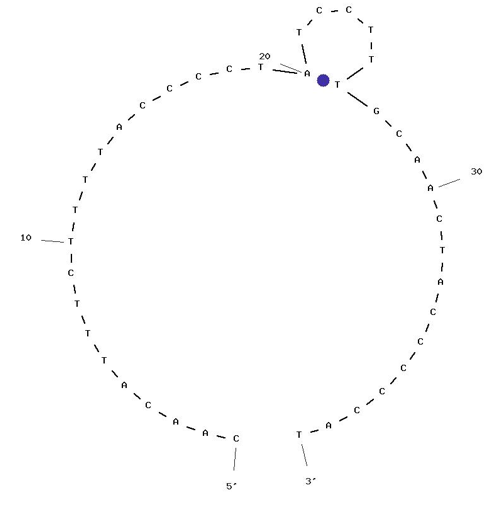


**Figure S1.** Prediction of the secondary structure of the selected DNA aptamers SEQ1-SEQ8 (A-H, respectively). The prediction was performed by UNAFold software at IDT (Integrated DNA Technologies) website. The unit for ΔG is kcal/mole.


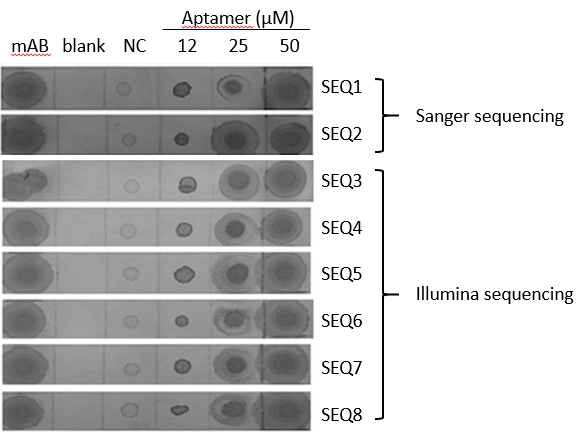


**Figure S2. Dot blot hybridization results**. The DNA aptamer candidates selected based on Sanger and Illumina sequencing analysis were evaluated for target binding capability using 5’ biotinylated synthesized oligonucleotides. 0, The chCD40_ED_ protein (15μg/mL) was fixed on an Immune-Blot PVDF membrane and incubated with the candidate aptamers at the indicated concentrations. Streptavidin added and then the BCIP/NBT substrate. The biotinylated anti-chicken CD40 mAb (10μg/mL) was included as the positive control (column A) and 1X imidazole phosphate buffer saline-tween (IPBST, column C) as a negative control.


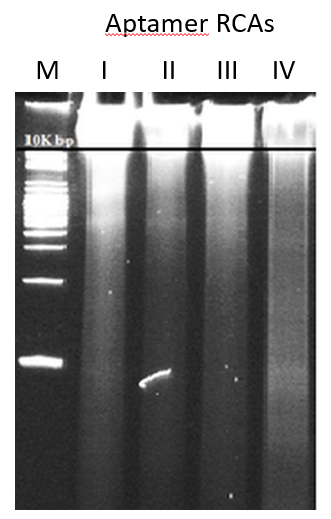


**Figure S3.** **RCA on denaturing 15%TBE urea gel**. According to the Illumina sequencing and Sanger sequencing, the complementary sequences of eight aptamers candidates suited to prepare four RCA templates. The highest two aptamers sequences represented in RCA I, and so forth. The templates flanked with primer binding site at the upstream and the downstream. Single primer used to amplify the RCA-p. The amplified RCA-p appear with very high molecular weight on the denaturing urea gel.


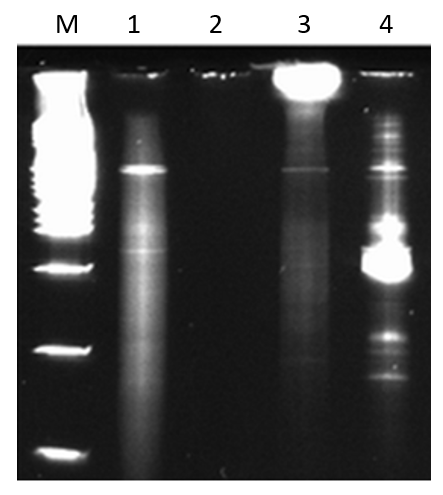


**Figure S4**. **RCA assessment with different patterns of digestion with AgeI restriction enzyme**. The linear single-stranded template DNA containing spacer region was circularized by primer and ligation with T4 DNA ligase. After the incubation with ɸ 29 DNA polymerase, the resulted RCA products were purified and precipitated. Spacer complementary sequence annealed thermally.

M: 2-log DNA ladder 1: Circularized template, 2: blank, 3: aptamer RCA II, 4: aptamer RCA digested with Age I


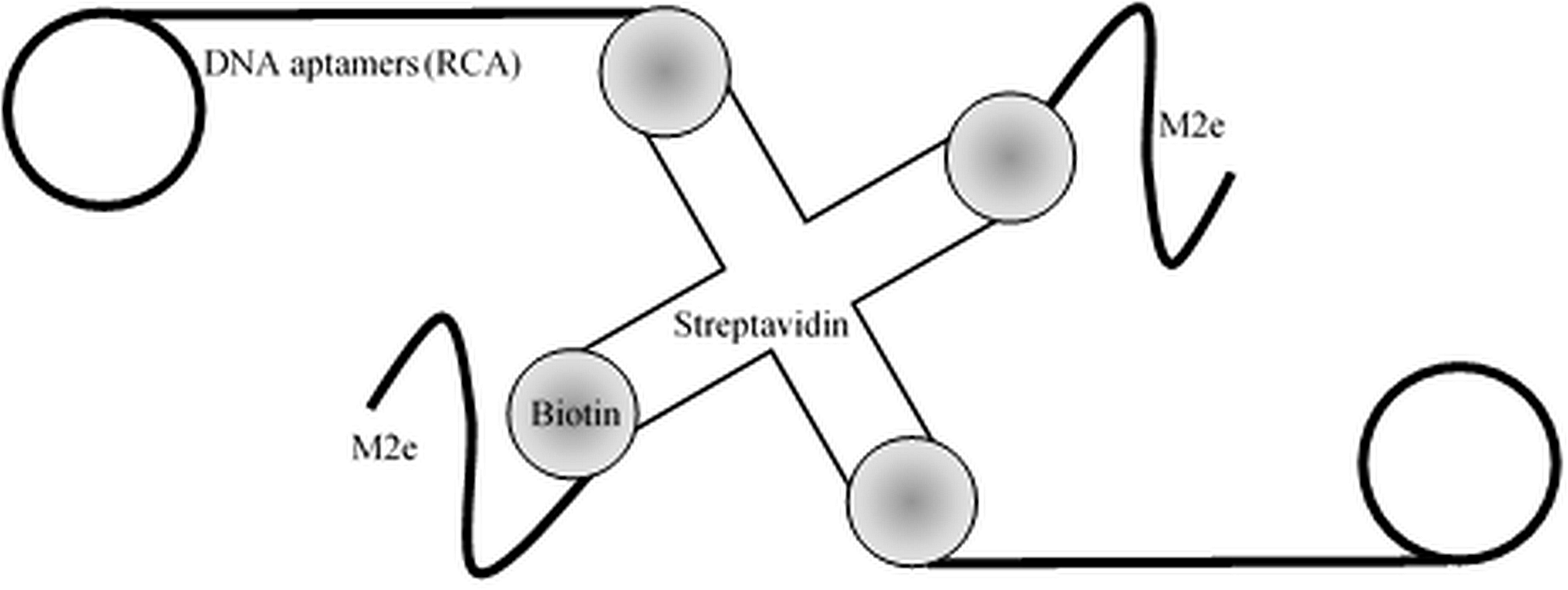


**Figure S5. Schematic diagram illustrating the design of the vaccine complex**. Streptavidin (SA) is the central molecule that holds the biotinylated components of the vaccine tightly. The biotinylated aptamer RCA and biotinylated M2e peptide were mixed together at 1:1 molar ratio, which was then mixed with SA at 2:1 molar ratio to form conjugated vaccine complex. This design principle was according to Chen et al. 2010.

**Additional Experiments for Characterization of SEQ3 and SEQ4**

**Results and Discussion**

**The structure of the aptamers.** Guanine-rich DNA and RNA molecules are known to fold into higher order structures ^[1]^. The most common of them are the inter- and intra-molecular structures called G-quadruplexes ^[2]^. Far UV circular dichroism spectroscopy (CD) is a valuable technique to characterize the secondary structures of nucleic acids ^[3]^. In this context, we measured the far UV CD spectra (200 nm – 320 nm) to characterize the structure and stability of SEQ3 and SEQ4 aptamers. Far UV CD spectrum of SEQ3 aptamer shows positive peaks at ~ 216 nm and 280 nm and negative bands centered at around 240 nm (Figure S6). In addition, a minor positive hump can also be discerned at 260 nm in the spectrum of SEQ3 aptamer. On the other hand, SEQ4 aptamer exhibits positive ellipticity bands at 220 nm and 285 nm and a negative peak at around 268 nm (Figure S6). B-DNA molecule with regular Watson-Crick base pairing will be expected to show a negative ellipticity band at ~ 240 nm and a positive band at ~ 260 nm ^[3]^. The fact, that both SEQ3 and SEQ4 aptamers exhibit CD bands centered around 285 nm, is indicative of the presence of G-tetrads/quadruplexes ^[4]^. In general, the CD bands in the 280 nm - 290 nm range are indicative of the stacking interactions of neighboring G‐tetrads with the same or opposing polarities of hydrogen bond acceptors and donors ^[4]^. Therefore, observation of the positive and negative bands at ~ 285 nm and ~268 nm, respectively in SEQ4 is suggestive of the predominant presence of antiparallel G-quadruplex folds(s) ^[5]^. Interestingly, the prominent negative band at ~ 240 nm and a minor positive peak and shoulder at 285 nm and 260 nm suggests that plausibly SEQ3 aptamer adopts a mixed parallel /antiparallel topology containing neighboring G-tetrad(s) of mixed relative polarities ^[4]^.

** Figure S6.** Overlay of the far-UV CD spectra of the aptamers. SEQ 3 at 25°C (blue) and SEQ 4 at 25°C (orange).

**The stability of the aptamers.** As the 285 nm band is a signature of the G-tetrads, we monitored the thermal stability of both SEQ3 and SEQ4 aptamers based on the changes in the intensity of the 285 nm peak in the temperature range of 30 °C – 95 °C. Figure S7 shows that intensity at 285 nm almost remains constant, for both SEQ3 and SEQ4 aptamers in the entire range of temperature. These results clearly indicate the SEQ3 and SEQ4 aptamers adopt an extremely stable three-dimensional structure. The extraordinary stability can be ascribed to the presence of a number of G-tetrads in both aptamers.

**Figure S7.** CD-monitored thermal denaturation profiles of aptamers. (Molar ellipticity values at 285 nm as a function of temperature). SEQ 3 (blue) and SEQ 4 (orange).

**Binding affinity of the aptamers to proteins present in a lipid environment.** Isothermal titration calorimetry (ITC) is a versatile technique that can measure the binding affinity of aptamers to their targets. We unfortunately could not obtain chCD40ED protein in large quantities to execute ITC experiments to measure the specific binding of SEQ3 and SEQ4 aptamers to chCD40ED protein. However, we performed ITC experiments to examine if the aptamers exhibit no-specific binding to proteins in a membrane environment. In this context, we measured the binding affinity of the SEQ3 and SEQ4 aptamers to bovine serum albumin (BSA) in the presence of 1,2-Dioleoyl-*sn*-glycero-3-phospho-*rac*-(1-glycerol) sodium salt (DOPG) liposomes. The isothermograms characterizing the titration showed that both the aptamers have very low or no binding affinity to BSA in the presence of the lipid vesicles (Figure S8, Panels B & C). In contrast, positive control experiments with the human acidic fibroblast growth factor (hFGF1) showed that the protein binds to its ligand (heparin) strongly (Figure S8, Panel A). Therefore, the ITC data show that the SEQ3 and SEQ4 aptamers do not exhibit non-specific binding to other proteins present in a lipid environment.

**Figure S8.** Isothermograms representing the titration of DOPG+BSA with the DNA aptamers (SEQ 3 and SEQ 4). wtFGF1 vs heparin (Panel – A), SEQ 3 vs DOPG+BSA (Panel – B), and SEQ 4 vs DOPG+BSA (Panel – C). The ITC data have been corrected for the heats of dilution.

**Materials and Methods**

**Far-UV CD data**. Circular dichroism spectra were performed using a Jasco J-1500 spectrophotometer. 1 mg/mL of aptamers (SEQ 3 and SEQ 4) were added to 10 mM PB (pH – 7.2) + 100 mM NaCl and loaded into a quartz cuvette of 0.1 cm path length. CD machine was kept at a constant temperature at 27⁰C. The wavelength of the spectrophotometer was set over the range of 200-320 nm and the spectra were scanned at 10 nm/min speed.

**Equilibrium unfolding experiment.** Thermal denaturation studies were performed on the JASCO-1500 spectrofluorometer using circular dichroism scans to reveal how temperature affects the stability of SEQ 3 and SEQ 4. 1 mg/mL of aptamers were added to 10 mM PB (pH – 7.2) + 100 mM NaCl and loaded to a 10 mm path length quartz cuvette and a temperature probe was inserted to monitor the temperature. Data were collected at 5⁰ C intervals over a temperature range of 25 ⁰C – 95 ⁰C. Data was plotted using Microsoft Excel as molar ellipticity at 260 nm vs temperature.

# Isothermal titration calorimetry. ITC was utilized to calculate the binding affinity of aptamers (SEQ 3 and SEQ 4) to DOPG + BSA. ITC measures the molar ratio of bound ligand (lipid + BSA) to the DNA aptamers at specified aliquots of titrant, which in this case is DNA. Within an adiabatic chamber, a series of 30 titrations were performed at 25°C with a stir speed of 700 rpm. DOPG and BSA was dissolved in 10 mM sodium phosphate buffer (pH – 7.2) containing 100 mM NaCl. The micelle formed was sonicated and the debris settled at the bottom was removed using micro pipette. The aptamers were prepared in 10 mM sodium phosphate buffer (pH – 7.2) containing 100 mM NaCl. 40 µM of DNA was titrated into 400 µM of (lipid+BSA) solution to achieve a final ratio of 1:10 (DNA: lipid+ protein). Data was plotted using Origin software and excess heats of dilution given from the reaction were appropriately subtracted out.

**References**

1. Sen, D. & Gilbert, W. Formation of parallel four-stranded complexes by guanine-rich motifs in DNA and its implications for meiosis. Nature. **334**, 364-366. (1988).
2. Han, H. & Hurley, LH. G-quadruplex DNA: a potential target for anti-cancer drug design. Trends Pharmacol. Sci. **21**, 136-142. (2000).
3. Patil, SD. & Rhodes, DG. Influence of divalent cations on the conformation of phosphorothioate oligodeoxynucleotides: a circular dichroism study. Nucleic Acids Res. **28**, 2439- 2445. (2000).
4. Zhou, B, *et al.* Characterizations of distinct parallel and antiparallel G-quadruplexes formed by two-repeat ALS and FTD related GGGGCC sequence. Sci. Rep. **8**, 1-7. (2018).
5. Gray, D. M. *et al*. Measured and calculated CD spectra of G-quartets stacked with the same or opposite polarities. Chirality **20**, 431–440 (2008).
